# KSNM 60 years: International Collaboration with Asian Nuclear Medicine Community

**DOI:** 10.1007/s13139-021-00692-9

**Published:** 2021-04-02

**Authors:** Jun Hatazawa

**Affiliations:** 1 Japan Radioisotope Association, Tokyo, Japan; 2 Research Center for Nuclear Physics, Osaka University, Osaka, Japan

On behalf of the people in the nuclear medicine community in Asia, I sincerely congratulate the 60th Anniversary of the Korean Society of Nuclear Medicine, and also the new editorship of the Official Journal of the KSNM, “Nuclear Medicine and Molecular Imaging”, by Prof. Hee-Seung Henry Bom. I witnessed the big wave of molecular imaging in Korea during the past 20 years. It is really a great success based on molecular biology as described in the Editorial article of “Short essay on 60 years’ challenges and achievements of KSNM” written by Drs. HG Ryoo, M Suh, JC Paeng, and JK Chung [1]. We all cerebrate the great achievement of the KSNM.

People in nuclear medicine community are happy because of its scientific base and clinical origin. The first medical application of radioisotope was Iodine-131 to treat a patient with hyperthyroidism on 31st March 1941 in Boston by Dr. Saul Hertz and his colleagues, followed by the treatment of thyroid cancer in 1946. This was a joint project of Harvard Medical School, Massachusetts General Hospital, Massachusetts Institute of Technology, and University of California Berkeley. The year of 2021 is the 80th Anniversary of medical use of radioisotopes. From the beginning, nuclear medicine directed a therapy, and nowadays we call it “Theranostics”. Integration of nuclear physics, nuclear chemistry, accelerator technology, production and purification of radioisotope met the clinical demands in Boston. Nuclear medicine impacted molecular biology and medicine first in vitro (radio-immunoassay) and now in vivo (molecular imaging). Eighty years later after the first use, we are returning to a therapy for a patient beyond the thyroid diseases and beyond the use of β emitting ^131^I.

Asian people in nuclear medicine community are happy because of the leadership of the KSNM and its members. The World Congress of Nuclear Medicine and Biology was held in 2006 in Seoul under the Chairmanship of Prof. MC Lee. The ARCCNM was launched in the early 2000s, and directed by Prof. MC Lee, Prof. JK Chung, and Prof. HS Bom. A number of the human resource development activities for nuclear medicine professionals in Asia were provided under the collaboration with International Atomic Energy Agency. The KSNM and its members are the engine of promoting nuclear medicine practice in our region. I am really proud of the achievement of the KSNM and its members.

Japanese people in nuclear medicine community are so happy because of the stimulation and encouragement from the KSNM members. I had an opportunity of the lecture during the 49th Annual Autumn Meeting of the KSNM in 2010. The topic of my talk was “Small budding of the PET/MR development and the accelerator-based boron neutron capture therapy (BNCT) with ^18^F-FBPA PET. Prof. JS Lee and Prof. SJ Hong continuously gave their ideas and guides on the PET/MR project. It was highlighted by “Nature” in 2009. The PET/MR is now further developed for free-radical imaging based on the Overhauser MRI (OMRI) [2]. The accelerator-based BNCT with ^10^B-boronophenylalanine (^10^B-BPA), a molecule targeting large amino acid transporter 1 (LAT1), predominantly expressed on cancer cells, was approved in 2020 for the treatment of intractable head and neck cancers after 10 years of effort into clinical trial [3]. The key issue in the BNCT is how to increase ^10^B concentration in tumors and to keep low in normal tissue. In the future BNCT, we need a molecular biology technique such as codon-optimized human sodium iodine symporter [4]

New faces of radioisotopes, molecules, and imaging instrument are waiting for clinical use. As mentioned in the Essay, ^223^Ra opened a door for the use of α emitting particle in medicine. The element 85, Astatine, is one of the candidates because of similar pharmacokinetics to β emitting ^131^I. ^211^At can be produced by the accelerator [5]. Beyond thyroid diseases, ^211^At-phenylalanine [6] and ^211^At-α-methyl tyrosine [7] of “cancer cell” specific LAT1 molecules were radiopharmaceuticals to treat a variety of human cancers by combining with “cancer stroma cell” targeting molecules [8]. The α particle imaging instrument is developed by combining “pinhole collimator” for low energy X-ray and Compton camera for high energy gamma-ray [9]. I learned the merits of “pinhole collimator” from the article of Prof. YW Bahk and his lecture in AOCNMB 2015 in Jeju [10].

Korean people and Japanese people in nuclear medicine community are happiest to have so many intellectual and personnel exchange for long time. I welcomed Prof. MC Lee, Prof. JK Chung, Prof. SE Kim, Prof. HS Bom, Prof. JT Lee, Prof. DH Moon, Prof. DS Lee, Dr. IY Hyun, Prof. KH Lee, Prof. JJ Min, and Prof. MJ Yun in Japan. I visited all of them during the past 10 years. Dr. IY Hyun stayed 1 year in Osaka and trained our staff.

We facilitate an exchange of academic stimulation and friendship in our region. Our patients are waiting for the progress and distribution of our specialty in the region during the next 60 years.

## References

[1] Ryoo HG, Suh M, Paeng JC, Chung J-K (2021). Short essay on 60 years’ challenges and achievements of KSNM. Nucl Med Mol Imaging.

[2] Yamamoto S, Watabe T, Ikeda H, Kanai Y, Ichikawa K (2016). Development of a PET/OMRI combined system for simultaneous imaging of positron and free radical probes for small animals. J Med Phys.

[3] Hirose K, Konno A, Hiratsuka J, Yoshimoto S, Kato T (2021). Boron neutron capture therapy using cyclotron-based epithermal neutron source and borofalan (10B) for recurrent or locally advanced head and neck cancer (JHN002): An open-label phase II trial. Radiother Oncol.

[4] Kim YH, Youn H, Na J, Hong KJ, Kang KW, Lee DS (2015). Codon-optimized human sodium iodine symporter (opt-hNIS) as a sensitive reporter and efficient therapeutic gene. Theranostics.

[5] Watabe T, Kaneda-Nakashima K, Liu Y, Shirakami Y, Ooe K (2019). Enhancement of ^211^At uptake via the sodium Iodide symporter by the addition of ascorbic acid in Targeted a-therapy of thyroid cancer. J Nucl Med.

[6] Watabe T, Kaneda-Nakashima K, Shirakami Y, Liu Y, Ooe K (2020). Targeted alpha therapy using astatine (^211^At)-labeled phenylalanine: a preclinical study in glioma bearing mice. Oncotarget.

[7] Kaneda-Nakashima K, Zhang Z, Manabe Y, Shimoyama A, Kabayama K, et al. a-emitting cancer therapy using ^211^At-AAMT targeting LAT1. Cancer Sci. 2020. 10.1111/cas.14761. 10.1111/cas.14761 PMC7935802 33277750

[8] Watabe T, Liu Y, Kaneda-Nakashima K (2020). Theranostics Targeting fibroblast activation protein in the tumor stroma: ^64^Cu- and ^225^Ac-labeled FAPI-04 in pancreatic cancer xenograft mouse models. J Nucl Med.

[9] Omata A, Kataoka J, Fujieda K, Sato S, Kuriyama E (2020). Performance demonstration of a hybrid Compton camera with an active pinhole for wide-band X-ray and gamma-ray imaging. Sci Rep.

[10] Bahk YW, Kim OH, Chung SK (1987). Pinhole collimator scintigraphy in differential diagnosis of metastasis, fracture, and infection of the spine. J Nucl Med.

